# Who are the convoys of the happiness of Chinese urban residents? Research on social relations and subjective well-being based on the convoy model

**DOI:** 10.3389/fpsyg.2023.1260754

**Published:** 2023-09-05

**Authors:** Jianna Li, Bin Guo, Mengyuan Lu, Wen Zhang

**Affiliations:** ^1^Department of Management Science and Engineering, School of Management, Xi'an University of Architecture and Technology, Xi'an, China; ^2^Department of Public Administration, School of Public Administration, Xi'an University of Architecture and Technology, Xi'an, China

**Keywords:** subjective well-being, the convoy model, social relations, kinship, friendship, neighborhood

## Abstract

**Introduction:**

While the rapid advancement of urbanization has driven the improvement of material living standards, it has also brought about rapid social changes and intensified competition. In this “involutive” environment characterized by highly competitive and strong pressure, urban residents tend to fall into a state of “mental exhaustion.” Anxiety, depression, sleep disorders, and other mental illnesses have seriously threatened public health in Chinese cities. Support from social relations is crucial for enhancing residents’ subjective well-being (SWB) and promoting their mental health, especially in China’s highly contextualized collectivist culture.

**Methods:**

According to the social structure of China’s “difference sequence pattern,” this paper constructs a theoretical framework of the relationship between social relations and SWB based on the convoy model and uses CGSS2018 data to verify the applicability of the theoretical framework.

**Results:**

Kinship and friendship positively relate to SWB, and their interaction effect is significantly negative. There is no necessary correlation between neighborhood and SWB. The relationship between social relations and SWB of different age groups is heterogeneous. In addition, the moderating effects of relative income and social class are significantly negative.

**Discussion:**

Kinship and friendship are Chinese urban residents’ SWB convoys, and these two factors have an obvious substitution effect. The neighborhood has withdrawn from the convoy orbit of Chinese urban residents’ SWB, which may be related to neighborhood indifference caused by China’s housing system reform. From the life course perspective, the SWB convoys of young and middle-aged groups consist of kinship and friendship, while those of elderly people include kinship and neighborhood. In addition, for poor individuals living at the bottom of society, support from kinship is the most important source of social capital. These findings provide new insights into the relationship between social relations and the welfare of Chinese urban residents.

## 1. Introduction

The continuous advancement of urbanization and the improvement of economic development have driven the rapid improvement of the living standards of urban residents in developing countries. However, the accompanying problems include the rapid transformation of society and the intensification of social competition caused by multiple challenges, such as the population expansion and resource pressure faced by modern cities (Guan et al., 2018). The related increasing social uncertainties and changing external environments cause people to live in a constant state of oppression and tension. Urban residents are gradually becoming introverted and closed-minded in regard to their work life and social life (Zhang and Ji, 2023). In this “involutive” environment characterized by highly competitive and strong pressure, people tend to indulge in their own stress, struggle to handle their negative emotions, and fall into a state of “mental exhaustion. “As a result, such an environment has a serious impact on the mental health and well-being of residents. Anxiety, depression, sleep disorders, and other mental illnesses have become major diseases threatening the health of urban residents (Fan et al., 2018). In this context, the necessity of studying the well-being of urban residents to promote their mental health is becoming increasingly prominent.

For China, the situation is not optimistic. According to the White Paper on Mental Health of Urban Residents in China, there are approximately 830 million urban residents in China, of which 610 million residents (accounting for 73.6% of the total urban population) are in a state of submental health, and 130 million residents are suffering from serious mental illnesses such as depression and anxiety, with a prevalence rate of mental disorders as high as 16.1%.^1^ Mental illness has become a serious threat to public health in Chinese cities. The happiness and mental health of urban residents are not only related to the quality of life for individuals and families but also have a crucial impact on not only the harmony and stability of the whole social economy but also the development level of cultural morality (Jackson, 2012). Therefore, understanding the factors that influence the well-being of urban residents and enhance their happiness to better promote their mental health is a major challenge that China must address.

Previous studies have shown that income only partially explains the subjective well-being (SWB) of Chinese people (Ding et al., 2021; Hu et al., 2021; Li and Zhao, 2022; Yang et al., 2022), while other factors affecting SWB remain unclear. Abundant evidence shows that support from social networks is positively correlated with SWB (Litwin, 2000; Powdthavee, 2008; Zhu et al., 2013). The convoy model of social relations suggests that over the life course of individuals, interactions with significant others in the social network have long-term and stable characteristics. The relationships evolve, and through the accumulation of interactions, these relationships gradually form part of individuals’ support network, providing social support and serving as “convoys” of their health and SWB (Antonucci et al., 2010, 2014).

However, what serve as the convoys of SWB for Chinese urban residents? This is an interesting issue in China. Traditional China is a typical relational society, with social networks mainly based on blood and geography being not only the basis for the operation of all social institutions and power but also an important source of emotional and instrumental support for people (Bian, 1997; Yang, 2005; Bian and Zhang, 2013). However, with accelerated urbanization in China, the dramatic increase in the scale of population mobility (taking the scale of urban household mobility as an example, the scale of urban household mobility in China increased by 192.66% in 2020 compared to 2010) and other changes in the urban social structure (Wang, 2021) may change the impact of intimate relationships constructed based on blood and geography on SWB. In this context, there is still a lack of research on how the SWB convoys of Chinese urban residents may change. What relationships exist among these convoys that affect people’s SWB? Little is known about these issues in the existing research.

In addition, there may be cultural differences in the association between social relations and SWB (Antonucci et al., 2010). From the spatiotemporal dimension, social relationships in Eastern and Western China have distinct characteristics. The logical starting point of Western social interaction is a “loose relationship,” namely, a relationship with a short duration in regard to the temporal dimension and high fluidity in regard to the spatial dimension; in contrast, the logical starting point of social relations in China is a “fixed relationship,” namely, a relationship with a long duration in regard to the temporal dimension and low fluidity in regard to the spatial dimension (Zhai, 2020). This difference in logical starting point may lead to variations in the mechanisms through which social relations affect SWB. For example, Fiori and colleagues found that compared to a selection of older adults from the United States, a similar sample of Japanese individuals evidenced no well-being differences by network type (Fiori et al., 2008). Although existing studies on social relations and SWB have made a series of achievements, most of them are based on the culture and context of Western societies, lacking relevant discussions on the highly contextualized social structure characteristics in China. Some studies that focus on the relationship between social networks and SWB in China tend to target specific age groups (Sun and Zhang, 2021) and thus lack a heterogeneous analysis of SWB convoys across age groups based on the perspective of life course characteristics related to social support. Moreover, China’s rapid rate of urbanization has brought about a widening wealth gap and an increase in social stratification; thus, what are the implications for SWB convoys? The existing research remains unclear on these issues.

Therefore, based on the convoy model of social relations and using 2018 CGSS data for empirical analysis with Chinese urban residents as samples, this study examines the relationship between residents’ social relations and SWB under the Chinese social and cultural background. It aims to offer countermeasures and suggestions for improving the social support network of Chinese urban residents and provide theoretical support for government agencies to formulate welfare policies and continuously enhance national SWB.

Compared with previous studies, this paper attempts to enrich the literature on the subject by answering the following questions. First, in an era of increasing marketization and economic rationality, who serve as the convoys of the SWB of Chinese urban residents? In other words, which groups of social support are more conducive to improving the SWB of Chinese urban residents? Second, what are the interaction effects between these convoys? The existing research is mostly based on the impact of social support group types on people’s SWB, while less attention is given to the relationships between these specific groups. This study attempts to investigate whether there are “substitution effects” among convoys, that is, whether other groups play a substitutive role in promoting their SWB when residents lack social support from a certain group. Third, in the social context of the wealth gap and social stratification, how do the socioeconomic characteristics of residents, such as relative income and social class, moderate the relationship between these convoys and SWB? Fourth, social relations reflect the nature of the life course; that is, the social relationship of individuals may change over time (Antonucci et al., 2014). For Chinese urban residents, are there differences in SWB convoys among different age groups? To this end, the following section reviews the relevant literature and proposes the research hypotheses. Section 3 presents the database, variables, and methods used in the empirical analysis. In Section 4, the hypotheses are tested, and the empirical results are reported. The research results are discussed in Section 5. Finally, the paper closes with a summary of the main results found in this study.

## 2. Literature review and research hypotheses

### 2.1. SWB and the convoy model

The country’s and public’s attention to “happiness” has promoted research on happiness economics in academic circles. Generally speaking, well-being can be divided into two dimensions: subjective and objective. The subjective dimension emphasizes the subjective experience of well-being, while the objective dimension focuses on the objective reality of well-being (Qi et al., 2023). Compared with objective dimensions, SWB has attracted extensive attention in the academic community due to its greater inclusiveness. Scholars have defined and explained the concept of SWB based on different theories and disciplines. After synthesizing the definitions of happiness by many Western philosophers and social scientists, Diener et al. concluded that SWB consists of three elements: life satisfaction, positive emotions, and negative emotions (Diener et al., 2003). The former belongs to the cognitive component, while the latter two belong to the emotional branch. This is one of the most widely accepted frameworks in hedonistic studies. Under this framework, scholars construct a conceptual framework about SWB regarding the life cognitive and emotional state dimensions (Keyes, 2006; Katsumi et al., 2021; Li et al., 2023; Zhou et al., 2023). Among them, the life cognition dimension is widely used because of its convenience, visualization, and practicality in assessing the SWB of different countries or cultures in large-scale surveys (Suh et al., 1998; Stone and Mackie, 2013). Therefore, this paper refers to the hedonistic view of SWB and focuses primarily on the life cognition dimension of SWB, considering that SWB is an individual’s comprehensive self-evaluation of their overall life status, satisfaction, and happiness.

Following the famous “happiness paradox” proposed by economist Easterlin (Easterlin, 1974), many scholars have paid much attention to SWB and its determinants. Personality characteristics, psychological factors, economic income, and other factors can explain the main differences in SWB. However, the social environment still significantly impacts personal SWB (Helliwell and Putnam, 2004; Sarracino, 2010; Neira et al., 2019), among which social relations play a nonnegligible role in residents’ SWB (Haller and Hadler, 2006). Social relations can promote SWB by providing people with emotional, instrumental, and other support (Diener, 2009; Putnam, 2015; Webster et al., 2021; Cheng et al., 2023).

Unlike other researchers’ exploration of the structure of social relations and the social support functions they provide, Kahn and Antonucci attempted to explore and explain individuals’ social relations and their impact on SWB from a developmental perspective through the convoy model (Kahn, 1980). The convoy model defines the boundaries of the social support network according to the closeness of the individual’s social relations over their lifespan of development. Social relations with high emotional intimacy with individuals occupy a closer convoy orbit, while social relations with moderate or low emotional intimacy with individuals are in a more detached convoy orbit (Antonucci and Akiyama, 1987). In social interaction, individuals exchange social support with social relations in different convoy orbits to help them cope with difficulties and pressures, improve their quality of life, and escort their SWB.

In previous research on applying the convoy model, scholars mainly focused on individuals’ “variable-centered” social support network to describe the composition of the convoy orbit and the exchange of their social relationships (Antonucci et al., 2010). In recent years, pioneers in the study of the convoy model have summarized the traditional research focus on convoys while reaffirming the connotations in the survey related to the convoy model, intending to complement and refine the analytical framework of the convoy model. One of the critical concerns is the delimitation of intimate social relations that as “convoys” (Antonucci et al., 2014). Specifically, relationships’ “depth” and “breadth” attributes have essential implications for the distribution of social relationships in an individual’s convoy orbit and for SWB. The size of social relations, such as the number of social companions, is considered a “breadth” attribute, which largely affects the distribution and total number of social relations in different convoy orbits (Antonucci et al., 2010). The strength of social relations, such as the frequency of contact with social peers, characterizes the “depth” attribute, which reflects the affective function of social support in the convoy model and is significantly associated with an individual’s SWB (Antonucci et al., 2014). Therefore, grasping the connotations of social relations is crucial for analyzing the structural and functional characteristics of SWB convoys.

**Figure 1:**
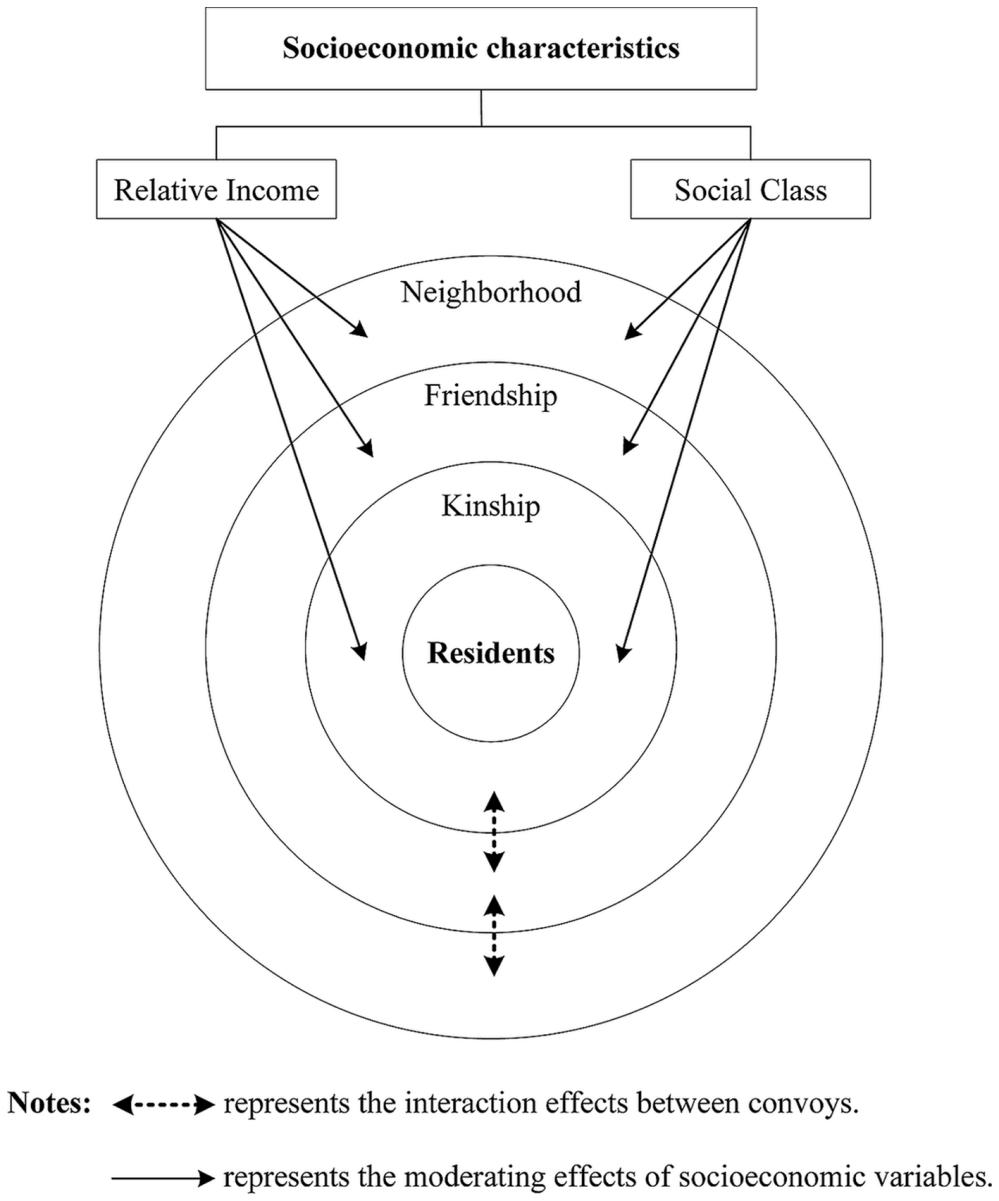
The theoretical framework of social relations and SWB based on the convoy model.

Chinese people pay more attention to “strong ties” constructed based on blood, kinship, and geography (Bian and Huang, 2015). Because the traditional social structure of China is the “difference sequence pattern” (Fei, 2019), that is, taking individuals as the center and spreading their relationship system outward to form “circles.” Close relationships with frequent interactions are usually in the “inner circle” (Yang, 2005), and the closer the association is to the center, the greater the influence on people’s lifestyles and SWB (Li and Luo, 2012). In line with this, the convoy model of social relations emphasizes that convoys have different relationship types and hierarchical characteristics. Based on the intimacy of their relationships and the independence of their social roles, three circles are divided: the core circle, the middle circle, and the outer circle, with the individuals residing in the center of these circles (Antonucci et al., 2014). Given this, and to enrich the relevant literature, this study provides a theoretical framework of social relations and SWB based on the convoy model, as shown in Figure 1. In this framework, based on the social structure of China’s “difference sequence pattern,” this paper delimits the distributional boundaries of different social relations (kinship, friendship, and neighborhood) in the convoy orbit and analyzes their effects on residents’ SWB, to deconstruct the connotations of the relationship between the convoys and the residents’ SWB from the depth attributes of the social relations. Furthermore, the interaction effects between these convoys are included in the framework to explore whether there are substitution effects between these convoys in promoting residents’ SWB, which goes beyond the neglect of relationships between convoys in previous studies. In addition, relative income and social class, two typical factors that characterize the economic and social status of residents, may cause differences in their social ties and patterns of interaction by shaping their social comparisons and social status, thus moderating their ability to obtain SWB from the support of their social networks. Therefore, the framework also considers the moderating effects of relative income and social class in the relationships between convoys and residents’ SWB. The framework and the theoretical assumptions based on it are specifically discussed below.

### 2.2. The theoretical framework of social relations and SWB based on the convoy model and research hypotheses

As shown in Figure 1, the core circle consists of the relationships that provide the most social support, usually including kinship based on blood (Antonucci et al., 2010). Kinship is more stable, and its intimacy is less affected by changing individual social roles. Relatives and individuals have high consistency in family networks and the growth experience. They share similar family perceptions, behavior patterns, and values. They tend to form higher interpersonal trust and reciprocity during interactions (Demir et al., 2018). Kinship can provide individuals with practical help and instrumental support and promote their SWB (Bian and Huang, 2015). Based on the above discussion, the following hypotheses are proposed:

*H1*: Kinship is the convoy of urban residents’ SWB; that is, kinship has a significant positive impact on urban residents’ SWB.

The middle circle usually involves friendship (Sun and Zhang, 2021). Compared with the core circle, friendship lacks the antecedent attribute of blood, which makes the intimacy of the relationship lower, and relationships may change with the development of the individual life course and social role. Thus, the stability of the relationship is somewhat lower (Demir et al., 2013). Friends often share similar values, personalities, or lifestyles, which can provide more tolerance, acceptance, understanding, and respect for individuals (Demir and Weitekamp, 2007). Close interactions with friends are an essential social support resource that helps individuals enhance their emotional pleasure (Greco et al., 2015). Based on the above analysis, this study proposes the following hypotheses:

*H2*: Friendship is the convoy of urban residents’ SWB; that is, friendship has a significant positive impact on urban residents’ SWB.

Compared with the relationships in the first two circles, the social relationships in the outer circle have the lowest degree of intimacy. However, they still provide individuals with necessary social support resources. In China, an old saying is that a close neighbor is better than a distant relative. Neighborhoods based on geopolitical factors can provide individuals with social support at the community level (Liu, 2016). Due to their proximity, neighborhoods can provide immediate information and resources about local life and immediate help when individuals encounter urgent problems (Ross et al., 2019). In addition, close neighborhood interaction can help individuals alleviate negative emotions and improve their life status (Yu et al., 2021), enhancing their life satisfaction. Based on this, the following hypothesis is proposed:

*H3*: Neighborhoods serve as a convoy of urban residents’ SWB; that is, neighborhoods have a significant positive impact on urban residents’ SWB.

Differences in the provision of social support by kinship, friendship, and neighborhood (Liebler and Sandefur, 1998), as well as individuals’ social networks, sociability, and personality traits, may influence their dependence on and choice of social relationships (Alonso, 2012). Close and enduring kinship, for example, can often provide individuals with life or financial support but may require the maintenance of specific expectations and role constraints (Hamberger et al., 2011), causing the individual to feel restricted in certain ways. In contrast, friendship is unrestricted and can provide emotional support and advice, providing new perspectives and ideas to help individuals face new challenges (Oishi, 2010). In this case, the boost of friendship to happiness may have a substitution effect on kinship. In addition, changes in individuals’ specific circumstances may also facilitate the substitution effect of SWB convoys (Thoits, 1995). For example, individuals may be estranged from relatives due to relocation, departure or financial reasons. In this situation, such individuals may turn their attention to friendship or their neighborhood for more social support, thus achieving relational substitution. Based on this, the following hypotheses are proposed:

*H4*: There is a significant substitution effect among kinship, friendship, and neighborhood in promoting residents’ SWB.

Specifically, we hypothesize as follows:

*H4-1*: There is a significant substitution effect between kinship and friendship in promoting residents’ SWB. In other words, the lower the frequency of one’s interaction with relatives is, the greater the positive impact of intimate connections with one’s friends is on residents’ SWB, and vice versa.

*H4-2*: There is a significant substitution effect between kinship and neighborhood in promoting residents’ SWB. In other words, the lower the frequency of one’s interaction with relatives is, the greater the positive impact of intimate connections with neighbors is on residents’ SWB, and vice versa.

*H4-3*: There is a significant substitution effect between friendship and neighborhood in promoting residents’ SWB. In other words, the lower the frequency of one’s interaction with friends is, the greater the positive impact of intimate connections with one’s neighbors is on residents’ SWB, and vice versa.

In addition, with the widening wealth gap and the intensification of social stratification, the socioeconomic characteristics of individuals may lead to differences in resources, status, and social support among individuals (Wang et al., 2005), which in turn have an impact on their SWB convoys, among which relative income and social class are two important factors. Relative income and social class may affect individuals’ roles and positions in their social networks (Antinyan et al., 2019), thereby affecting the method and content of the support provided by their convoys. Individuals with lower relative income or social class may have relatively weaker ties in their social networks; thus, they may cherish and rely more on their social networks to meet their basic needs (Letki and Mieriņa, 2015) to gain a sense of happiness, such as obtaining economic assistance, sharing resources, and social emotional support. Individuals with higher relative income and social class, on the other hand, have more resources themselves (Behtoui and Neergaard, 2012); thus, they are relatively less dependent on support from social networks to sustain their SWB. Based on this, the following hypotheses are proposed:

*H5*: Relative income can significantly and negatively moderate the contribution of social relations to residents’ SWB.

Specifically, we hypothesize as follows:

*H5-1*: Relative income can significantly negatively moderate the promoting effect of kinship on residents’ SWB.

*H5-2*: Relative income can significantly negatively moderate the promoting effect of friendship on residents’ SWB.

*H5-3*: Relative income can significantly negatively moderate the promoting effect of neighborhood on residents’ SWB.

*H6*: Social class can significantly and negatively moderate the contribution of social relations to residents’ SWB.

Specifically, we hypothesize as follows:

*H6-1*: Social class can significantly negatively moderate the promoting effect of kinship on residents’ SWB.

*H6-2*: Social class can significantly negatively moderate the promoting effect of friendship on residents’ SWB.

*H6-3*: Social class can significantly negatively moderate the promoting effect of neighborhood on residents’ SWB.

## 3. Materials and methods

### 3.1. Data source

The data used in this study are from the Chinese General Social Survey (CGSS), a nationally representative continuous cross-sectional census started in 2003 to collect a wide range of socioeconomic data and analyze the quality of life of Chinese people. This paper uses data from the most recent round (2018), which covers 12,787 urban and rural residents over 18 years old in all provinces, cities, and autonomous regions of mainland China. We selected 9,104 urban residents, excluded respondents with missing relevant data (such as rejection, inapplicability, or ignorance), and finally collected a sample of 5,712 urban residents for empirical analysis.

### 3.2. Variables

#### 3.2.1. Dependent variable

The dependent variable in this study is SWB, denoted by SW. As defined above, this study measured SWB through perceptions of life dimensions. The CGSS questionnaire is measured by answers to “Generally, do you think your life is happy?” with responses on a scale ranging from 1 to 5 (where 1 = very unhappy and 5 = very happy). Although it is relatively simple in measurement, this method has the advantage of psychological surveying and can reflect the respondents’ true SWB to a greater extent (Veenhoven and Ehrhardt, 1995).

#### 3.2.2. Independent variable

The core explanatory variables of this study include kinship, friendship, and neighborhood. Kinship is measured in the CGSS by answering “Frequency of gathering with relatives who do not live together,” which uses a scale ranging from 1 to 5 (where 1 = never and 5 = every day). Friendship is measured by two questions in the CGSS: “Frequency of gathering with friends,” which is assigned a value of 1–5 according to its frequency from low to high, and “Frequency of social activities with friends,” which is set a value of 1–7 according to its frequency from low to high; the value of friendship is the average of these two scores. The neighborhood is measured in the CGSS by answering “Frequency of social activities with neighbors,” which uses a scale ranging from 1 to 7 (where 1 = never and 7 = almost every day).

In addition, referring to the operation methods of previous relevant studies (Rafnsson et al., 2015; Wang, 2016; Cheng et al., 2018; Vezzoli et al., 2023), we introduce other control variables that may affect SWB. At the level of essential demographic characteristics, these variables include gender, age, marital status, party membership, years of education, and health status. At the family economic level, these variables include absolute income, measured by annual family income, and relative income, measured by the local level of family financial status. At the social status level, these variables include social class, measured by the perception of one’s current social class; finally, at the macro social level, these variables include social trust and social equity. Table 1 summarizes the main variables used in this study and their descriptive statistical results.

**Table 1:** Main variables and descriptive statistical results.

Variables	Definition	Mean	SD
Dependent variable			
SW	Very unhappy, unhappy, neither happy nor unhappy, happy, and very happy (value: 1–5)	3.95	0.77
Core independent variables			
Kinship	Frequency of interaction with relatives (value: 1–5)	2.24	0.73
Friendship	Frequency of interaction with friends (value: 1–6)	3.31	1.17
Neighborhood	Frequency of interaction with neighbors (value: 1–7)	4.37	2.17
Control variables			
Age	Age from 18 to 93	51.38	16.33
Gender	Women: the value of 1; otherwise: the value of 0	0.49	0.50
Marital status: married	Married: the value of 1; otherwise: the value of 0	0.80	0.40
Marital status: divorced or widowed	Divorced or widowed: the value of 1; otherwise: the value of 0	0.12	0.32
Years of education	Years of education estimated according to the highest level of education achieved (value: 0–19)	14.26	4.46
Party membership	Party Member: the value of 1; otherwise: the value of 0	0.16	0.36
Health status	Very unhealthy, unhealthy, average, healthy, very healthy (value: 1–5)	3.68	0.99
Absolute income	The logarithm of annual household income (value: 4–17)	11.10	1.07
Relative income	Well below average, below average, average, above average, well above average (value: 1–5)	2.68	0.71
Social class	From the bottom to the top of society (value: 1–10)	4.43	1.58
Social trust	Very distrustful, distrustful, neither trustful nor distrustful, trustful, very trustful (value: 1–5)	3.55	0.99
Social equity	Completely unfair, unfair, neither fair nor unfair, fair, completely fair (value: 1–5)	3.18	1.00

CGSS 2018.

### 3.3. Methods

Since the dependent variable, SW, is ordered, this paper uses the ordered logit regression model for quantitative analysis, with particular attention to the role of social network support in SWB. The baseline regression equation is as follows; we set up Model 1 as shown in Equation (1) to test hypotheses H1–H3:

(1) $${\mathrm{SW}}_{j}=\beta X_{\mathrm{ij}}+\Pi C_{j}+\varepsilon_{j}$$

In Equation (1), the variable SW is an indicator to measure residents’ SWB, the variable *X* is an indicator to measure residents’ social relations, and β represents regression coefficients of social relation. *C* is a matrix of other control variables affecting residents’ SWB, and Π is the regression coefficient matrix of the corresponding control variables. The subscript i=1,2,3 denotes the *i*th social relation (kinship, friendship and neighborhood, respectively), and the subscript *j* denotes the *j*th observation sample. ε is a random disturbance term.

Based on Model 1, we set up Model 2 as shown in Equation (2) to test Hypothesis H4:

(2) $${\mathrm{SW}}_{j}=\beta_{1}X_{\mathrm{mj}}+\beta_{2}X_{\mathrm{nj}}+\beta_{3}X_{\mathrm{mj}}*X_{\mathrm{nj}}+\Pi C_{j}+\varepsilon_{j}$$

where m,n=i , $X_{m}*X_{n}$ represents the interaction term of the residents’ social relations. The addition of this interaction term can help us test the interaction effect between social relations. For example, if there is a significant negative interaction effect between $X_{\mathrm{mj}}$ and $X_{\mathrm{nj}}$ , it indicates that as the frequency of communication of $X_{m}$ decreases, the positive impact of $X_{n}$ on SWB gradually increases. This implies that in terms of SWB convoys, $X_{n}$ has a substitution effect on $X_{m}$ .

Next, we set up Model 3 as shown in Equation (3) to test H5 and H6:

(3) $${\mathrm{SW}}_{j}=\beta_{1}X_{\mathrm{ij}}+\beta_{2}M_{\mathrm{pj}}+\beta_{3}X_{\mathrm{ij}}*M_{\mathrm{pj}}+\Pi C_{j}+\varepsilon_{j}$$

In Equation (3), p=1,2 , M is the moderating variable of the relationship between social relations and residents’ SWB, and $M_{p}$ represents the *p*th moderating variable. By adding these interaction terms, Model 3 can help us analyze the moderating effects of relative income and social class on the relationships between social relations and SWB.

## 4. Results

### 4.1. Baseline regression results

First, we conduct a full-sample regression on the relationship between social network support and the SWB of Chinese urban residents, and the results are shown in Table 2. Column (1) shows the baseline model results, including only the control variables. Columns (2)–(4) show the regression results of the network support of kinship, friendship, and neighborhood on SWB, respectively. We observe that the coefficients of kinship and friendship are both significantly positive at the level of 0.1%, indicating that close interaction with relatives and friends can significantly improve the SWB of Chinese urban residents. We further calculate the average marginal effect of kinship and friendship on different levels of SWB, as shown in Table 3. We find that when other factors are held constant, the probability of residents feeling “very happy” increases by 2.05% for each unit increase in the frequency of interaction with relatives. Every unit increase in the frequency of interaction with friends increases the probability of residents feeling “very happy” by 1.04%. Therefore, hypotheses H1 and H2 are supported. The coefficient of the neighborhood does not pass the significance test at the 5% level. Therefore, for Chinese urban residents at the present stage, the frequency of interaction with neighbors has no significant effect on their SWB, and Hypothesis H3 is not supported.

**Table 2:** Impact of social relations on SWB (full-sample regression).

Variables	(1)	(2)	(3)	(4)
Age	−0.0471***	−0.0481***	−0.0467***	−0.0462***
	(0.0118)	(0.0117)	(0.0118)	(0.0118)
Age squared	0.000601***	0.000612***	0.000606***	0.000594***
	(0.000110)	(0.000110)	(0.000110)	(0.000111)
Gender	0.202***	0.195***	0.203***	0.206***
	(0.0557)	(0.0556)	(0.0557)	(0.0557)
Married	0.503***	0.505***	0.527***	0.506***
	(0.114)	(0.114)	(0.114)	(0.114)
Divorced or widowed	0.141	0.141	0.146	0.144
	(0.144)	(0.143)	(0.144)	(0.144)
Years of education	0.00255	0.00131	0.00285	0.00201
	(0.00647)	(0.00649)	(0.00649)	(0.00649)
Party membership	0.144*	0.136*	0.141*	0.140*
	(0.0768)	(0.0772)	(0.0769)	(0.0769)
Health status	0.379***	0.374***	0.372***	0.381***
	(0.0337)	(0.0337)	(0.0337)	(0.0337)
Absolute income	0.107***	0.0974***	0.101***	0.103***
	(0.0309)	(0.0309)	(0.0309)	(0.0311)
Social equity	0.504***	0.509***	0.507***	0.505***
	(0.0335)	(0.0334)	(0.0335)	(0.0335)
Relative income	0.282***	0.276***	0.273***	0.284***
	(0.0495)	(0.0495)	(0.0495)	(0.0496)
Social class	0.174***	0.170***	0.171***	0.175***
	(0.0217)	(0.0217)	(0.0217)	(0.0216)
Social trust	0.325***	0.324***	0.324***	0.326***
	(0.0334)	(0.0333)	(0.0334)	(0.0333)
Kinship		0.151***		
		(0.0399)		
Friendship			0.0770***	
			(0.0254)	
Neighborhood				−0.0154
				(0.0132)
*N*	5,712	5,712	5,712	5,712

Standard errors in parentheses. *p < 0.1, ***p < 0.01.

**Table 3:** Average marginal effects of core explanatory variables.

Variables	Very unhappy	Unhappy	Neither happy Nor unhappy	Happy	Very happy
Kinship	−0.00123***	−0.00651***	−0.0114***	−0.00138**	0.0205***
	(0.000370)	(0.00173)	(0.00301)	(0.000622)	(0.00539)
Friendship	−0.000628***	−0.00331***	−0.00579***	−0.000704**	0.0104***
	(0.000224)	(0.00110)	(0.00192)	(0.000342)	(0.00344)
Neighborhood	0.000126	0.000665	0.00116	0.000141	−0.00209
	(0.000109)	(0.000567)	(0.000989)	(0.000134)	(0.00179)

Standard errors in parentheses. **p < 0.05, ***p < 0.01.

The estimation results of other control variables are consistent with the literature (Lu et al., 2020; Aliyev et al., 2022). There is a U-shaped curve relationship between age and SWB; that is, the SWB of urban residents first decreases and then increases with age, which is consistent with the findings within the literature (Olivos et al., 2021). Compared with men, women have higher levels of SWB, which may be because men have more family and social responsibilities than women. The SWB of married people is higher than that of unmarried people, which means that marriage enriches the lives of urban residents, bringing them more happiness. Health status also significantly affects the SWB of urban residents. The higher the self-rated health status is, the higher the individual’s SWB is. Absolute income, relative income, and social class all significantly promote the SWB of urban residents. Both social trust and social equity also significantly contribute to the SWB of residents at the 1% level. The impacts of years of education and party membership on SWB are not significant.

### 4.2. Interaction effect regression results

Next, we analyze the interaction effects of social relations. To prevent multicollinearity caused by the interaction terms, we centralize the relevant variables and include the appropriate interaction terms in the regression model. The results are shown in Table 4. Column (1) includes the interaction item of kinship and friendship (Kinship X Friendship). The result suggests that the interaction item is significantly negative. In contrast, kinship and friendship positively and significantly impact SWB. This indicates that the less frequent the interaction with relatives is, the greater the positive effect of interaction with friends on residents’ SWB, and vice versa. The same is true of friendship’s impact on the relationship of kinship and SWB. Hence, Hypothesis H4-1 is supported. In Column (2), the result that includes the interaction between kinship and neighborhood (Kinship X Neighborhood) shows that the less frequent interaction is with neighbors, the greater the promoting effect of interaction with relatives on SWB, and vice versa. Therefore, Hypothesis H4-2 is only partially supported. Specifically, as a convoy of residents’ SWB, kinship has a substitution effect on neighborhood. However, one’s neighborhood cannot serve as a substitute for kinship in the promotion of residents’ SWB. Column (3) includes the interaction item of friendship and neighborhood (Friendship X Neighborhood), and the result shows that neighborhood has no significant interaction effect on friendship. Hypothesis H4-3 is not supported.

**Table 4:** The interaction effects of different social relations on SWB.

Variables	(1)	(2)	(3)
Age	−0.0473***	−0.0471***	−0.0440***
	(0.0117)	(0.0118)	(0.0118)
Age squared	0.000610***	0.000605***	0.000588***
	(0.000110)	(0.000110)	(0.000111)
Gender	0.196***	0.198***	0.216***
	(0.0557)	(0.0558)	(0.0559)
Married	0.523***	0.517***	0.549***
	(0.114)	(0.114)	(0.115)
Divorced or widowed	0.151	0.151	0.157
	(0.143)	(0.143)	(0.144)
Years of education	0.00172	0.000726	0.00152
	(0.00651)	(0.00651)	(0.00653)
Party membership	0.136*	0.130*	0.130*
	(0.0772)	(0.0775)	(0.0770)
Health status	0.370***	0.376***	0.373***
	(0.0337)	(0.0338)	(0.0337)
Absolute income	0.0919***	0.0912***	0.0867***
	(0.0308)	(0.0312)	(0.0315)
Social equity	0.510***	0.511***	0.510***
	(0.0334)	(0.0335)	(0.0335)
Relative income	0.273***	0.281***	0.276***
	(0.0495)	(0.0496)	(0.0496)
Social class	0.169***	0.171***	0.171***
	(0.0217)	(0.0217)	(0.0217)
Social trust	0.322***	0.324***	0.325***
	(0.0333)	(0.0333)	(0.0334)
Kinship	0.137***	0.165***	
	(0.0430)	(0.0405)	
Friendship	0.0453*		0.113***
	(0.0270)		(0.0285)
Neighborhood		−0.0231*	−0.0419***
		(0.0133)	(0.0148)
Kinship × friendship	−0.0540*		
	(0.0305)		
Kinship × neighborhood		−0.0400**	
		(0.0175)	
Friendship × neighborhood			−0.00204
			(0.0111)
N	5,712	5,712	5,712

Standard errors in parentheses. *p < 0.1, **p < 0.05, ***p < 0.01.

### 4.3. Moderating effect regression results

Next, we conduct a regression analysis of the moderating effects of relative income and social class on the relationships between social relations and SWB.^2^ The results are shown in Table 5. Columns (1)–(2) show the results of the moderating effects of relative income on kinship and friendship, respectively. According to the estimated results in Column (1), the relative income and kinship interaction term is significantly negative. In contrast, kinship and relative income have a positive and significant impact on SWB, indicating that with the increase in relative income, the promotion effect of support from kinship on individuals’ SWB decreases, and Hypothesis H5-1 is supported. The regression result in Column (2) shows that relative income has no significant moderating effect on friendship, and Hypothesis H5-2 is not supported. Columns (3)–(4) show the results of the moderating effect of social class on kinship and friendship, respectively. The regression result in Column (3) shows that social class significantly negatively moderates the promoting effect of kinship on SWB, indicating that the promotion effect of kinship on SWB decreases with the rise of the social class level of individuals. Hence, Hypothesis H6-1 is supported. Similarly, we do not observe a significant moderating effect of social class on friendship, and Hypothesis H6-2 is not supported.

**Table 5:** The moderating effects of relative income and social class on social relations and SWB.

Variables	(1)	(2)	(3)	(4)
Age	−0.0475***	−0.0464***	−0.0472***	−0.0463***
	(0.0117)	(0.0117)	(0.0118)	(0.0118)
Age squared	0.000607***	0.000602***	0.000603***	0.000601***
	(0.000110)	(0.000110)	(0.000110)	(0.000110)
Gender	0.199***	0.205***	0.195***	0.204***
	(0.0557)	(0.0557)	(0.0556)	(0.0557)
Married	0.503***	0.531***	0.504***	0.528***
	(0.114)	(0.115)	(0.114)	(0.115)
Divorced or widowed	0.135	0.152	0.135	0.145
	(0.143)	(0.144)	(0.143)	(0.144)
Years of education	0.000659	0.00261	0.000997	0.00283
	(0.00646)	(0.00648)	(0.00648)	(0.00648)
Party membership	0.142*	0.142*	0.140*	0.142*
	(0.0773)	(0.0769)	(0.0773)	(0.0769)
Health status	0.374***	0.370***	0.374***	0.370***
	(0.0338)	(0.0336)	(0.0337)	(0.0336)
Absolute income	0.0953***	0.101***	0.0958***	0.101***
	(0.0308)	(0.0309)	(0.0308)	(0.0309)
Social equity	0.508***	0.506***	0.508***	0.506***
	(0.0334)	(0.0334)	(0.0334)	(0.0335)
Relative income	0.273***	0.270***	0.279***	0.275***
	(0.0493)	(0.0495)	(0.0496)	(0.0496)
Social class	0.173***	0.172***	0.170***	0.169***
	(0.0217)	(0.0218)	(0.0217)	(0.0217)
Social trust	0.322***	0.324***	0.324***	0.324***
	(0.0333)	(0.0334)	(0.0333)	(0.0334)
Kinship	0.153***		0.153***	
	(0.0399)		(0.0401)	
Friendship		0.0751***		0.0744***
		(0.0254)		(0.0254)
RI X Kinship	−0.161***			
	(0.0572)			
RI X Friendship		−0.0508		
		(0.0357)		
SC X Kinship			−0.0554**	
			(0.0243)	
SC X Friendship				−0.0242
				(0.0159)
N	5,712	5,712	5,712	5,712

Standard errors in parentheses. *p < 0.1, **p < 0.05, ***p < 0.01.

### 4.4. Expanding analysis

**Figure 2:**
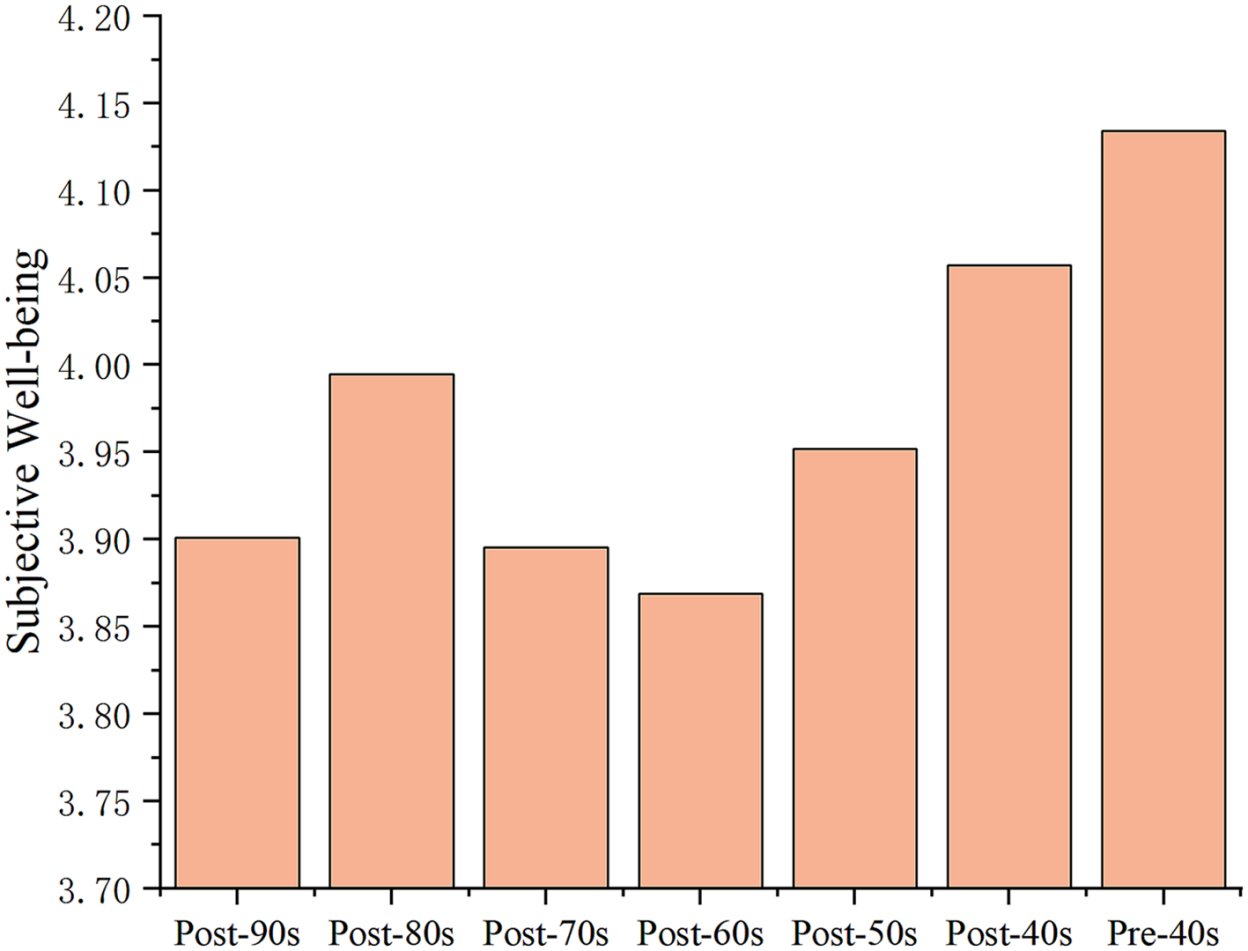
Histogram of the mean value of the SWB of residents in different generations in 2018. CGSS 2018.

The convoy model emphasizes the adoption of a lifelong developmental and life course perspective to depict the social network of individuals. It focuses on the changes in social relations that convoy an individual’s SWB over time at different life stages. Therefore, based on the above analysis, we expand our analysis of the heterogeneity in social relations across groups at the level of SWB. First, we divide the samples into generations, that is, individuals belonging to the same era (10 years) are divided into a group, thus obtaining seven groups of samples, namely, “post-90s,” “post-80s,” “post-70s,” “post-60s,” “post-50s,” “post-40s” and “pre-40s,” to observe the differences in happiness between them. Figure 2 illustrates the differences in the mean values of the SWB of residents across generations. The SWB of Chinese urban residents shows a wave trend of “ $\cap^{”}$ ” and then “ $\cup^{”}$ ” among different generational groups.

**Figure 3:**
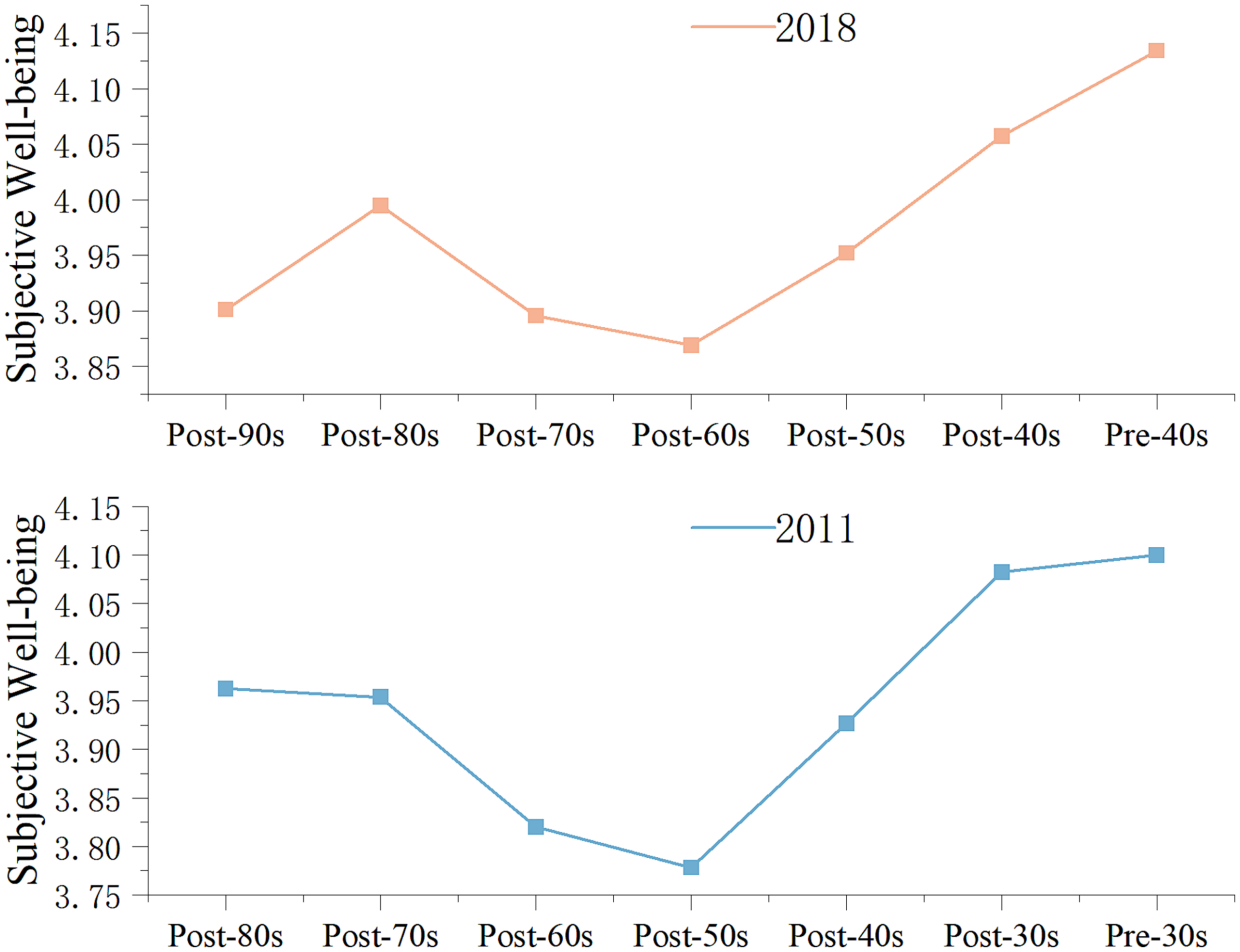
Comparison line graph of mean SWB of residents’ SWB between 2011 and 2018. CGSS 2018, 2011.

To verify whether the above trend of SWB difference is an accidental phenomenon or a rule caused by the different age stages between generations, relevant data from CGSS 2011^3^ are used for verification. For the “post-90s” residents, who were in the adolescent stage in 2011 and had not yet stepped out of the campus environment, the impact mechanism of their SWB is special; therefore, the data sample of the “post-90s” in 2011 is excluded. The “post-80s” residents in 2011 and the “post-90s” residents in 2018 are at similar age stages; thus, the “post-80s” residents in 2011 can be compared as the “reference group” of the “post-90s” residents in 2018, and so on. We plot a comparison line graph of the mean SWB of residents in different generations based on a “reference group” composed of residents of similar age in 2011 and 2018 (as shown in Figure 3). As shown in Figure 3, the differences in the mean SWB values of residents in different generations in 2011 and 2018 show approximately the same trend, indicating that the differences in SWB between generations show a certain regularity with age stage. According to Figure 3, and referring to Chun Luo’s suggestion on the division of age groups of the Chinese population at the present stage (Luo, 2017), we further divide the sample into four groups, young (corresponding to the “post-90s” and “post-80s” in 2018), middle aged (corresponding to the “post-70s” and “post-60s” in 2018), early old aged (corresponding to the “post-50s” and “post-40s” in 2018), and old aged (corresponding to the “pre-40s” in 2018)^4^. Combined with Figure 3, we can summarize the law of SWB of different groups as follows: the SWB began to decline after a small peak in the young group, and the middle-aged group’s SWB is in the trough, followed by the beginning of the early old-aged group’s SWB beginning to show an upward trend, and the old-aged group’s SWB is the highest.

Then, we conduct the regression analysis for each group to observe the heterogeneity of their SWB convoys, as shown in Table 6. The regression results of the young sample [Columns (1)–(3)] show that the coefficients of kinship and friendship are significantly positive at the level of 1%, and the results of the middle-aged sample [Columns (4)–(6)] show that the coefficient of kinship is significantly positive at the level of 1%. The coefficient of friendship is significantly positive at the level of 0.1%, which suggests that close interaction with relatives and friends can significantly improve the SWB of young and middle-aged people. According to the regression results in Columns (7)–(9), we do not observe a significant impact of network support from kinship, friendship, and neighborhood on the SWB of the early old age group. The regression results of the old-aged sample [Columns (10)–(12)] show that the kinship coefficient is significantly positive at the level of 1%. The neighborhood coefficient is significantly positive at 5%, indicating that for the old-aged group, close interaction with relatives and neighbors can significantly promote their SWB.

**Table 6:** Impact of social relations on SWB (grouping sample regression).

Variables	(1)	(2)	(3)	(4)	(5)	(6)
	Youth			Middle aged		
Age	−0.0992	−0.105	−0.100	−0.00595	−0.0179	0.000753
	(0.126)	(0.124)	(0.126)	(0.149)	(0.149)	(0.149)
Age Squared	0.00162	0.00176	0.00164	0.000210	0.000338	0.000155
	(0.00202)	(0.00201)	(0.00202)	(0.00151)	(0.00152)	(0.00152)
Gender	0.0937	0.108	0.107	0.212**	0.226**	0.218**
	(0.109)	(0.110)	(0.109)	(0.0906)	(0.0909)	(0.0908)
Married	0.643***	0.678***	0.653***	0.163	0.186	0.188
	(0.158)	(0.161)	(0.158)	(0.271)	(0.273)	(0.280)
Divorced or Widowed	−0.880**	−0.840**	−0.886**	−0.635**	−0.639**	−0.610*
	(0.347)	(0.347)	(0.345)	(0.307)	(0.310)	(0.315)
Years of Education	−0.0308	−0.0283	−0.0318	0.0236**	0.0246**	0.0234**
	(0.0199)	(0.0202)	(0.0197)	(0.0109)	(0.0110)	(0.0110)
Party Membership	0.0764	0.0790	0.0626	0.223	0.205	0.215
	(0.147)	(0.147)	(0.148)	(0.143)	(0.142)	(0.143)
Health Status	0.410***	0.401***	0.416***	0.367***	0.366***	0.373***
	(0.0774)	(0.0774)	(0.0771)	(0.0548)	(0.0543)	(0.0549)
Absolute Income	−0.0222	−0.0361	−0.0267	0.135***	0.132***	0.137***
	(0.0673)	(0.0674)	(0.0673)	(0.0512)	(0.0510)	(0.0513)
Social Equity	0.519***	0.524***	0.523***	0.495***	0.495***	0.493***
	(0.0722)	(0.0727)	(0.0722)	(0.0546)	(0.0544)	(0.0548)
Relative Income	0.147	0.136	0.155	0.324***	0.317***	0.334***
	(0.100)	(0.101)	(0.101)	(0.0838)	(0.0833)	(0.0838)
Social Class	0.172***	0.177***	0.179***	0.134***	0.134***	0.142***
	(0.0451)	(0.0452)	(0.0449)	(0.0365)	(0.0366)	(0.0365)
Social Trust	0.298***	0.301***	0.299***	0.347***	0.344***	0.352***
	(0.0637)	(0.0640)	(0.0639)	(0.0551)	(0.0549)	(0.0552)
Kinship	0.175**			0.171**		
	(0.0855)			(0.0671)		
Friendship		0.124**			0.132***	
		(0.0607)			(0.0450)	
Neighborhood			−0.0268			−0.0347
			(0.0274)			(0.0222)
*N*	1,535	1,535	1,535	2,155	2,155	2,155

Variables	(7)	(8)	(9)	(10)	(11)	(12)
	Early old aged			Old aged		
Age	0.101	0.109	0.113	1.795	2.281	2.394
	(0.248)	(0.248)	(0.248)	(2.010)	(1.971)	(2.004)
Age Squared	−0.000566	−0.000629	−0.000657	−0.0100	−0.0129	−0.0136
	(0.00182)	(0.00181)	(0.00181)	(0.0118)	(0.0116)	(0.0117)
Gender	0.191*	0.204**	0.208**	0.707**	0.672**	0.600*
	(0.101)	(0.101)	(0.101)	(0.324)	(0.325)	(0.325)
Married	0.879*	0.869*	0.865*	1.654**	1.648**	1.898**
	(0.501)	(0.496)	(0.497)	(0.774)	(0.764)	(0.806)
Divorced or Widowed	0.887*	0.879*	0.876*	1.626**	1.605**	1.902**
	(0.507)	(0.503)	(0.503)	(0.776)	(0.774)	(0.807)
Years of Education	−0.00298	−0.00200	−0.00258	−0.0162	−0.0155	−0.0152
	(0.00952)	(0.00949)	(0.00954)	(0.0212)	(0.0212)	(0.0210)
Party Membership	0.0772	0.0908	0.0878	0.381	0.383	0.368
	(0.128)	(0.128)	(0.128)	(0.330)	(0.336)	(0.335)
Health Status	0.363***	0.368***	0.370***	0.410**	0.404**	0.410**
	(0.0542)	(0.0547)	(0.0543)	(0.171)	(0.173)	(0.173)
Absolute Income	0.131**	0.137***	0.134**	0.291**	0.330**	0.357***
	(0.0529)	(0.0527)	(0.0531)	(0.131)	(0.135)	(0.136)
Social Equity	0.501***	0.497***	0.498***	0.822***	0.778***	0.779***
	(0.0552)	(0.0555)	(0.0555)	(0.197)	(0.185)	(0.186)
Relative Income	0.285***	0.291***	0.291***	0.348	0.363	0.333
	(0.0838)	(0.0839)	(0.0837)	(0.259)	(0.257)	(0.255)
Social Class	0.224***	0.225***	0.226***	0.0944	0.0770	0.0868
	(0.0371)	(0.0371)	(0.0370)	(0.112)	(0.111)	(0.110)
Social Trust	0.324***	0.324***	0.326***	0.258	0.304	0.335*
	(0.0598)	(0.0599)	(0.0599)	(0.187)	(0.190)	(0.192)
Kinship	0.0847			0.350**		
	(0.0668)			(0.165)		
Friendship		−0.00649			0.133	
		(0.0396)			(0.101)	
Neighborhood			−0.0143			0.109*
			(0.0220)			(0.0614)
*N*	1789	1789	1789	233	233	233

Standard errors in parentheses. *p < 0.1, **p < 0.05, ***p < 0.01.

We further calculate the average marginal effects of social relations for each group, as shown in Table 7. The results show that while keeping other factors constant, the probability of feeling “very happy” increases by 2.33% for young residents, 2.01% for middle-aged residents, and 5.57% for old-aged residents, with each increase of one unit of interaction frequency with relatives. For every unit increase in the frequency of interaction with friends, the probability of feeling “very happy” increases by 1.64% for young residents and 1.55% for middle-aged residents. Each increase in the frequency of interaction with neighbors increases the probability of the old-aged sample feeling “very happy” by 1.73%. This means that close interaction with relatives has an enormous marginal effect on the SWB of the old-aged group, and close interaction with friends has a great marginal impact on the SWB of the young group. The estimated results of other control variables do not change significantly, and we will not repeat them here.

**Table 7:** Average marginal effects of social relations for each group.

Group	Variables	Very unhappy	Unhappy	Neither happy Nor unhappy	Happy	Very happy
Youth	Kinship	−0.00101*	−0.00586**	−0.0145**	−0.00192	0.0233**
		(0.000581)	(0.00290)	(0.00713)	(0.00154)	(0.0113)
	Friendship	−0.000711*	−0.00413**	−0.0103**	−0.00134	0.0164**
		(0.000409)	(0.00207)	(0.00508)	(0.00106)	(0.00804)
	Neighborhood	0.000154	0.000896	0.00222	0.000298	−0.00357
		(0.000166)	(0.000917)	(0.00227)	(0.000366)	(0.00365)
Middle aged	Kinship	−0.00161**	−0.00810**	−0.0139**	0.00351**	0.0201**
		(0.000727)	(0.00320)	(0.00547)	(0.00167)	(0.00788)
	Friendship	−0.00125**	−0.00625***	−0.0107***	0.00269**	0.0155***
		(0.000499)	(0.00214)	(0.00368)	(0.00116)	(0.00529)
	Neighborhood	0.000328	0.00165	0.00283	−0.000718	−0.00409
		(0.000218)	(0.00107)	(0.00181)	(0.000491)	(0.00262)
Early old aged	Kinship	−0.000731	−0.00387	−0.00521	−0.00297	0.0128
		(0.000604)	(0.00308)	(0.00413)	(0.00239)	(0.0101)
	Friendship	0.0000561	0.000297	0.000399	0.000227	−0.000980
		(0.000344)	(0.00181)	(0.00244)	(0.00139)	(0.00598)
	Neighborhood	0.000124	0.000654	0.000878	0.000499	−0.00215
		(0.000194)	(0.00101)	(0.00135)	(0.000776)	(0.00332)
Old aged	Kinship	−0.00283	−0.0108*	−0.0167*	−0.0254*	0.0557**
		(0.00239)	(0.00589)	(0.00852)	(0.0115)	(0.0250)
	Friendship	−0.00105	−0.00419	−0.00644	−0.00965	0.0213
		(0.00114)	(0.00351)	(0.00524)	(0.00682)	(0.0159)
	Neighborhood	−0.000861	−0.00342	−0.00524	−0.00778*	0.0173*
		(0.000830)	(0.00212)	(0.00325)	(0.00422)	(0.00953)

Standard errors in parentheses. *p < 0.1, **p < 0.05, ***p < 0.01.

## 5. Discussion

### 5.1. Discussion of research results

Through frequent interactions with individuals in the social network, people can obtain particular social support and emotional satisfaction, which is very important for maintaining SWB. However, there are cultural differences in the impact of social network support on SWB, which highlights the importance of studying social support groups in different cultural backgrounds. It is interesting to discuss this issue in the context of Chinese culture because the social networks of urban residents in China have been regulated for a long time under the influence of traditional culture and are constantly evolving with economic growth and urbanization. Therefore, based on the convoy model, this paper focuses on the impact of social relations on the SWB of Chinese urban residents. Combined with the existing relevant literature, we discuss the empirical research results as follows.

First, in general, the research results validate the applicability of the theoretical framework of social relations and SWB based on the convoy model proposed in this paper. Social relations based on depth attribute connotation can significantly improve the SWB of urban residents. This means that the strong ties theory still applies to modern Chinese urban society (Bian and Huang, 2015). For urban residents in a high-pressure social environment, strong ties with close interaction can improve their living conditions and enhance their SWB of overall life by providing necessary social support, such as helping them find jobs (Bian, 1997), providing economic support (Li and Luo, 2012), and emotional assistance (Bian and Zhang, 2013). In addition, our results also show a certain regularity in SWB among different generational groups, with the middle-aged group having the lowest SWB. It confirms the situation of the middle-aged group as the “sandwich” generation when facing multiple roles and pressures (Miller, 1981). Compared with other groups, middle-aged people often need to deal with their career development, children’s upbringing and education and care for elderly parents, and other family responsibilities simultaneously, bearing the dual pressure of family and career, playing a sandwich role. This significantly reduces their subjective perception of happiness in life (Gillett and Crisp, 2017). In this case, our results suggest that close interactions with relatives and friends can help the middle-aged group cope with various challenges in life and significantly improve their SWB.

Second, from the perspective of the convoy role of the residents’ SWB, kinship can significantly enhance the SWB of Chinese urban residents, serving as the most important convoy of SWB for young, middle-aged, and old-aged groups. This outcome is consistent with Fei’s view on the social structure of the traditional “difference sequence pattern” in China (Fei, 2019). This result suggests that in the modern era of rapid economic and social development, kinship constructed on the basis of blood is still an unbreakable interpersonal bond and plays an important social support role for modern Chinese people. Our results also indicate that the social support obtained from one’s blood relatives has the largest marginal effect on SWB enhancement in the old-aged group. This may be because elderly people are more deeply influenced by the traditional Chinese concept of “ethical orientation” (Cheng et al., 2009), and their social interaction habits show a trend of involution (Peng, 2021). In interpersonal communication, elderly individuals pay more attention to the sense of security and happiness brought about by kinship based on blood ties.

Friendship serves as a SWB convoy for the young and middle-aged groups in urban China. This result supports the view of Demir et al. (Demir et al., 2015; Manago and Vaughn, 2015). To a certain extent, the above mentioned result reflects the social network changes that occurred with regard to middle-aged and young people. The growth of population mobility and social heterogeneity has increasingly pushed urban residents into secondary interpersonal relationships within a “stranger society” (Lu and Qian, 2019). Especially for young and middle-aged people, friendships based on common interests and similar values help them build self-esteem and gain not only emotional support but also a sense of pleasure (Hunagund and Hangal, 2014). In addition, instrumental support such as rich heterogeneous information conveyed by friends can also help these individuals cope with and alleviate the pressures brought about by social changes and the accelerated pace of life (Hällsten et al., 2017); thus, friendships with long duration and high fluidity are increasingly important in the lives of young and middle-aged people in urban China.

While there is no necessary correlation between neighborhood and the SWB of all Chinese urban residents, one’s neighborhood does serve as a SWB convoy for those in the old-aged group. This result is consistent with the view of Howley et al. that the relationship between neighborhood interaction and life satisfaction may vary from person to person (Howley et al., 2015). Although geographically constructed neighborliness used to be an important part of traditional Chinese social networks (Fei, 2019), due to the reform of the urban housing system that has occurred in China, commercial housing communities have replaced unit-based communities; such reform has led to a gradual fracture in the traditional close neighborhood relationship (Li and Cai, 2020). For most urban residents, the indifference of one’s neighborhood dramatically weakens the importance of neighborhood support (Xu and Zheng, 2022). However, elderly people have previously experienced close emotional and helpful neighborhood relationships and thus still attach importance to neighborhood interaction. Most elderly individuals can maintain good neighborhood relationships and obtain stable social support from neighborhood interactions (Sun and Zhang, 2021).

Third, from the perspective of the relationship between convoys of residents’ SWB, there is a significant substitution effect between kinship and friendship with regard to serving as SWB convoys of Chinese urban residents. Schnettler and Wöhler demonstrated the alternative mechanisms of friend networks for the social support of childless elderly people in the West from the perspective of network size (Schnettler and Wöhler, 2016). Based on the traditional Chinese perspective of strong relationships, our study verifies the substitution effect of kinship and friendship regarding the promotion of residents’ SWB; that is, if people lack support from their good friends, then their relatives become particularly important to their SWB, and vice versa. In addition, since the neighborhood’s main effect on SWB is insignificant, it has no substitute effect on kinship and friendship. Once residents lack the support of kinship and friendship, they cannot obtain enough social support from their neighbors to help them maintain SWB.

Fourth, from the perspective of the impact of individuals’ socioeconomic characteristics on the convoy model of SWB, both relative income and social class significantly negatively moderate the escort effect of kinship on residents’ SWB. This result shows that the promoting impact of kinship on the SWB of low-income people (or those with lower social classes) exceeds the promoting effect on the SWB of high-income people (or those with higher social classes). This means that for the poor living at the bottom of society, support from kinship is the most important source of social capital, confirming the view that kinship is still the “capital of the poor” for modern urban Chinese residents (Grootaert, 1999).

### 5.2. Implications

Based on the above findings and the corresponding discussion, this paper proposes several suggestions to enhance residents’ SWB by improving their social support network. First, the findings highlight the importance of kinship in maintaining the SWB of Chinese urban residents. Therefore, in rapidly developing urban environments with high population mobility and social changes, the government should strengthen the education of kinship values, promote traditional culture and guide residents to attach importance to harmonious family and kinship relationships. In addition, the establishment of kinship guarantee mechanisms and constraining mechanisms may be an effective strategy for enhancing kinship and reducing conflict.

Second, policymakers can build more social platforms for middle-aged and young people by organizing cultural and sports activities, public welfare activities, or volunteer teams, which will promote their SWB by expanding their social networks.

Third, when improving the social support system for urban residents in China, the government should pay special attention to the “short board” of the neighborhood. Community social support is mainly bred in the neighborhood and can provide timely, comprehensive, and practical assistance to residents. Our results indicate that the neighborhood is particularly important for maintaining the SWB of elderly people. Therefore, urban managers can repair neighborhoods by strengthening community management and services, carrying out mutual help-based activities, promoting community culture and improving the living environment to promote the SWB of residents, especially elderly individuals.

Fourth, policymakers should also pay attention to the low-income groups living in cities. Because the social support systems of those in this group are relatively weak, when they do encounter difficulties, the members of this group will be in trouble if their relatives cannot provide help. Therefore, the government should take relevant measures, such as establishing a comprehensive public welfare system, providing social salvage and assistance, improving the community network service system, providing employment and training opportunities, etc., to expand the social support networks of low-income groups and prevent the sudden decline in their happiness index caused by a rupture in their single source of social support.

### 5.3. Limitations and future research directions

We should point out the limitations of this study. When analyzing the differences in social support networks of different groups, we find that for the early old age group (60–79 years old), kinship, friendship, and neighborhood cannot significantly improve their SWB. This finding is inconsistent with the conclusions of existing studies on the SWB of elderly individuals. There are two possible reasons for this. One is the age division of the population sample. Existing studies on the elderly mostly divide people over 60 into elderly samples. Considering the current situation of the Chinese population structure and the health status of elderly individuals, we separate the “young elderly” aged 60–79 from the overall sample of elderly individuals to more clearly observe the differences in intergenerational social network support. They often have no evident decline in physical and psychological functions, in contrast to the assumptions of previous studies (Luo, 2017). This may have contributed to the difference in the results because the conclusions of our study on the old age sample (older than 80) are consistent with those of existing studies (Sun and Zhang, 2021; Xie and Lu, 2022). Second, most early old age people are in retirement, which may cause significant changes in their social network structure, including the termination of many relationships (Zhang et al., 2022) (especially with colleagues) and changing the function of their social network (Antonucci et al., 2006). Research shows that retirees are inclined to receive less support and give more support to others to strengthen their self-reliance ability and compensate for the loss of fulfillment caused by unemployment (Antonucci, 2001). This may be one of the reasons for the particular social support network of the early old age group. Due to space limitations, this paper does not conduct a more in-depth analysis of the samples of the early old age group, which is the main direction for further research.

## 6. Conclusion

Social relations are crucial to people’s SWB, especially in the context of China’s highly contextualized collectivist culture. However, with the rapid progress of urbanization and the constant changes that are made in regard to social structure, which social relations are the most important with regard to serving as SWB convoys for urban residents in China? What are the relationships that exist among these convoys? How do socioeconomic characteristics affect the role of these relations in convoying SWB? From the perspective of the individual life course, are there differences in the SWB convoys of people at different stages of life? The existing research on these issues is still unclear. Focusing on the structural characteristics of Chinese society, this study responds to the above questions based on the convoy model, which enriches the literature to a certain extent. Through the empirical analysis of micro survey data drawn from the 2018 CGSS, the conclusions of this study are as follows.

First, kinship is the most important SWB convoy for urban Chinese residents, especially for poor individuals living at the bottom of the hierarchy, for whom kinship is the most important source of social support.

Second, friendship is also a SWB convoy of urban residents, and there is a significant substitution effect between friendship and kinship.

Third, there is no significant correlation between neighborhood and SWB, which may be related to the neighborhood indifference caused by China’s housing system reform.

Fourth, both relative income and social class significantly weaken the contribution of kinship to residents’ SWB.

Fifth, there is heterogeneity among different groups of SWB convoys. Specifically, the SWB convoys of young and middle-aged groups consist mainly of kinship and friendship, while those of elderly people mainly include kinship and neighborhood.

These findings provide new insights into the relationship between social relations and the welfare of Chinese urban residents and have implications for not only improving the social support network of Chinese urban residents but also continuously improving national SWB through intervention implementation.

## Data availability statement

Publicly available datasets were analyzed in this study. This data can be found at: http://cgss.ruc.edu.cn/index.htm.

## Author contributions

JL: Conceptualization, data curation, formal analysis, methodology, resources, software, validation, visualization, writing – original draft, writing – review & editing. GB: Methodology, project administration, supervision, writing – review & editing, funding acquisition. ML: Data curation, writing – review & editing. WZ: Data curation, writing – review & editing.

## Funding

The author(s) declare that no financial support was received for the research, authorship, and/or publication of this article.

## Conflict of interest

The authors declare that the research was conducted in the absence of any commercial or financial relationships that could be construed as a potential conflict of interest.

## Publisher’s note

All claims expressed in this article are solely those of the authors and do not necessarily represent those of their affiliated organizations, or those of the publisher, the editors and the reviewers. Any product that may be evaluated in this article, or claim that may be made by its manufacturer, is not guaranteed or endorsed by the publisher.

## Footnotes

^1^The data come from the White Paper on Mental Health of Chinese Urban Residents released by the Chinese Medical Association in 2018.

^2^As the main effect of neighborhood is not significant, Hypothesis H5-3 and H6-3 are not supported. Only the moderating effect of relative income and social class on kinship and friendship is analyzed here.

^3^It would be more accurate to use the relevant data of CGSS 2008 here; unfortunately, the content of the CGSS 2008 questionnaire is quite different from that of the CGSS 2018 questionnaire, making it impossible to obtain the comparative data. Therefore, we chose to use data from the next round after 2008, that is, the CGSS 2011 questionnaire.

^4^Since there are only 6 observation samples aged 18 years old, they are included in the youth group in order to simplify the analysis.
